# Differential physiological, transcriptomic and metabolomic responses of Arabidopsis leaves under prolonged warming and heat shock

**DOI:** 10.1186/s12870-020-2292-y

**Published:** 2020-02-22

**Authors:** Li Wang, Kai-Biao Ma, Zhao-Geng Lu, Shi-Xiong Ren, Hui-Ru Jiang, Jia-Wen Cui, Gang Chen, Nian-Jun Teng, Hon-Ming Lam, Biao Jin

**Affiliations:** 1 College of Horticulture and Plant Protection, Yangzhou University, Yangzhou, 225009 China; 2 Center for Soybean Research of the State Key Laboratory of Agrobiotechnology and School of Life Sciences, The Chinese University of Hong Kong, Hong Kong, SAR China; 3 College of Bioscience and Biotechnology, Yangzhou University, Yangzhou, 225009 China; 4 College of Horticulture, Nanjing Agricultural University, Nanjing, 210095 China

**Keywords:** *Arabidopsis thaliana*, Climate warming, Heat stress, Omics, Photosynthesis, Respiration, Transcription factors

## Abstract

**Background:**

Elevated temperature as a result of global climate warming, either in form of sudden heatwave (heat shock) or prolonged warming, has profound effects on the growth and development of plants. However, how plants differentially respond to these two forms of elevated temperatures is largely unknown. Here we have therefore performed a comprehensive comparison of multi-level responses of Arabidopsis leaves to heat shock and prolonged warming.

**Results:**

The plant responded to prolonged warming through decreased stomatal conductance, and to heat shock by increased transpiration. In carbon metabolism, the glycolysis pathway was enhanced while the tricarboxylic acid (TCA) cycle was inhibited under prolonged warming, and heat shock significantly limited the conversion of pyruvate into acetyl coenzyme A. The cellular concentration of hydrogen peroxide (H_2_O_2_) and the activities of antioxidant enzymes were increased under both conditions but exhibited a higher induction under heat shock. Interestingly, the transcription factors, class A1 heat shock factors (HSFA1s) and dehydration responsive element-binding proteins (DREBs), were up-regulated under heat shock, whereas with prolonged warming, other abiotic stress response pathways, especially basic leucine zipper factors (bZIPs) were up-regulated instead.

**Conclusions:**

Our findings reveal that *Arabidopsis* exhibits different response patterns under heat shock versus prolonged warming, and plants employ distinctly different response strategies to combat these two types of thermal stress.

## Background

As a result of climate warming, plants, due to their sessile lifestyle, need to develop a set of responses to adapt to the increasing temperature. Past studies on elevated-temperature treatments could be generalized into two categories: short-term intensive heat (also known as heat shock) and prolonged warming. Traditionally, for heat shock treatment, plants are subjected to a temperature that is much higher (such as 10–15 °C above ambient) than their optimal threshold within a very short time (from several minutes to a few hours) [1]. On the other hand, prolonged warming is normally simulated by exposing plants to a moderately elevated temperature (such as 2–5 °C above their optimal temperature range) for several days, weeks, or even the whole growing season [2, 3].

Previous studies on prolonged warming mainly investigated the phenology, reproduction and productivity, growth and development, and biomass accumulation [3–6], at the community, population and species levels, on species varying from grasses to trees [2, 7], while a few others examined the cellular, physiological and metabolomic responses [8–10]. However, the comprehensive analyses of responses to prolonged warming are rare.

In contrast, the physiological and molecular mechanisms involved in the responses to heat shock have been extensively studied in plants. Generally, heat shock reduces the photosynthetic and respiratory activities and diminishes productivity [9]. Intense heat induces structural and functional changes in the thylakoid membranes in photosynthetic apparatus, as a result of the production of reactive oxygen species (ROS) causing damage to a wide range of cellular components [11]. In response to heat shock, plants accelerate the production of heat shock proteins (HSPs) and accumulate responsive metabolites [12]. The roles of master transcriptional regulator HSFA1s and several other transcription factors, have been uncovered in the heat shock-related signaling pathways [13, 14]. However, very little is known about such regulatory mechanisms in prolonged warming responses. In particular, no study has focused on a systematic comparison of plant responses between prolonged warming and heat shock, despite the frequent occurrence of both in nature.

Leaves are the main vegetative organs that directly sense the changes in ambient temperature, and they can express phenotypically plastic responses to environmental temperature changes [15]. Moreover, leaf photosynthesis and transpiration, as the basis of plant growth and development, are susceptible to temperature changes [9]. Consequently, experiments on the effects of elevated temperatures on leaves will provide a better understanding of plant responses to heat stress. Therefore, we compared the physiological, transcriptomic and metabolomic responses of *Arabidopsis* leaves between prolonged warming and heat shock, and interpret these results in the contexts of their effects on photosynthesis and respiration, as well as the underlying transcriptional regulation.

## Results

### Physiological and biochemical changes

**Fig. 1:**
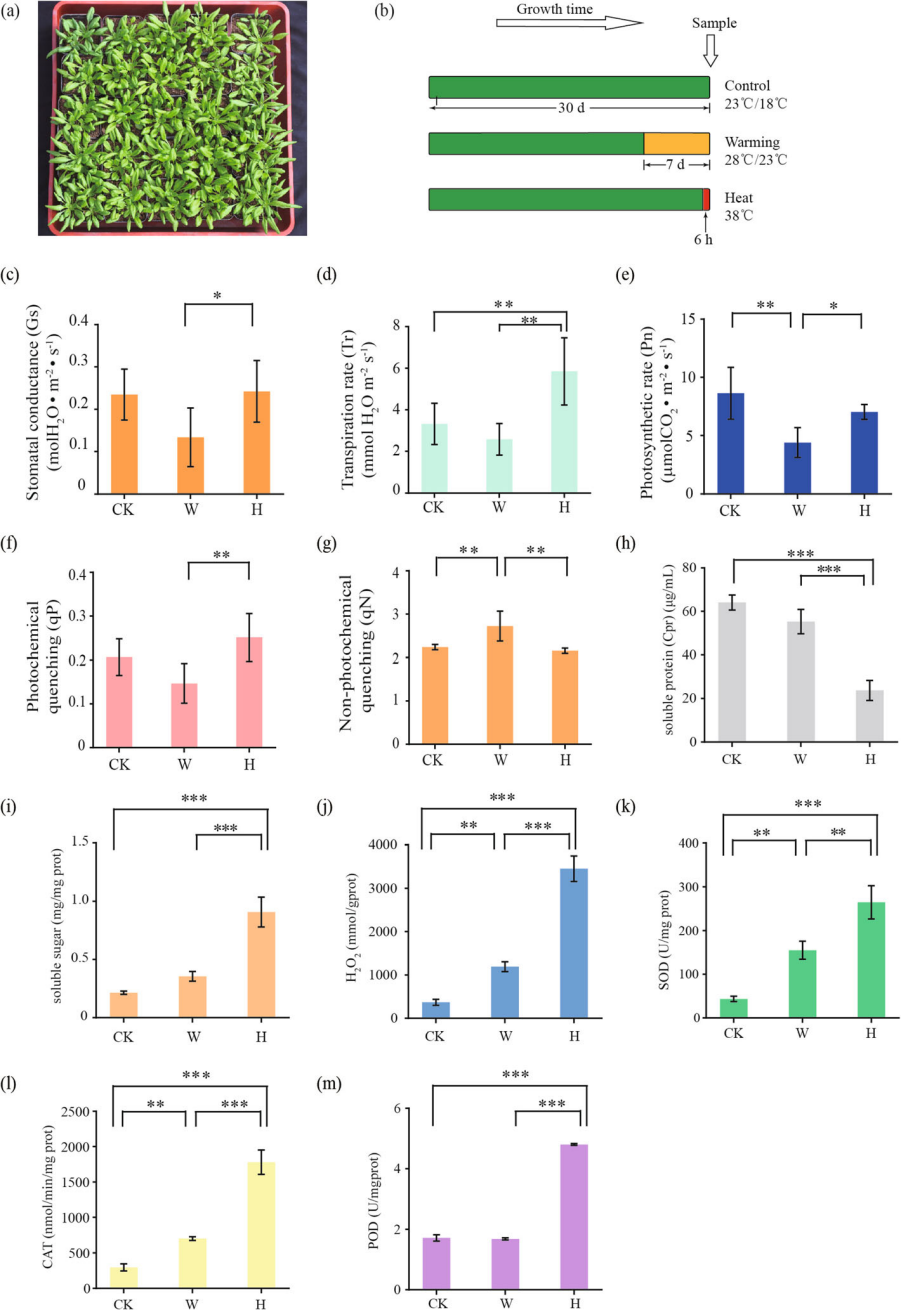
Physiological characteristics of *A. thaliana* under different elevated temperature treatments. **a** Typical *A. thaliana* plants at the rosette growth stage 30 days after sowing. **b** The timeline for control (CK), prolonged warming (PW) and heat shock (HS) treatments showing a relatively prolonged period of warming (orange) for 7 days and a short-term heat shock (red) for 6 h. **c** Stomatal conductance, (**d**) transpiration rate, (**e**) photosynthetic rate, (**f**) photochemical quenching (qP), and (**g**) non-photochemical quenching (qN) were measured with a LI-6400*XT* Portable Photosynthesis System. **h** Soluble proteins, (**i**) soluble sugars, (**j**) hydrogen peroxide, the activities of (**k**) catalase (CAT), (**l**) superoxide dismutase (SOD) and (**m**) peroxidase (POD) in the leaves were determined at the end of the elevated temperature treatments. CK: control; PW: prolonged warming; HS: heat shock. Error bars represent mean ± S.D. (c)-(g), *n* = 8, (**h-m**), *n* = 3, */**/***: *p* < 0.05/0.01/0.001, respectively

*Arabidopsis thaliana* plants were grown under control (CK), prolonged warming (PW) and heat shock (HS) treatments, and the leaves at the rosette growth stage were sampled (Fig. 1a, b). The stomatal conductance decreased under prolonged warming (Fig. 1c). However, the transpiration rate increased significantly under heat shock (Fig. 1d). Compared to control and heat shock, photosynthetic rate was decreased by prolonged warming (Fig. 1e). Similarly, prolonged warming had more pronounced effects on both qP and qN, by decreasing qP and raising qN (Fig. 1f, g). However, there is no significant difference in qP and qN between control and heat shock (Fig. 1f, g).

Compared to control and prolonged warming, the level of soluble proteins was significantly lowered in heat shock (Fig. 1h). On the other hand, heat shock produced a significantly larger increase in both soluble sugars and hydrogen peroxide than prolonged warming (Fig. 1i, j). The activities of superoxide dismutase (SOD), catalase (CAT) and peroxidase (POD) in heat shock were significantly higher than those in prolonged warming (Fig. 1k, l, m).

### Metabolite changes

**Fig. 2:**
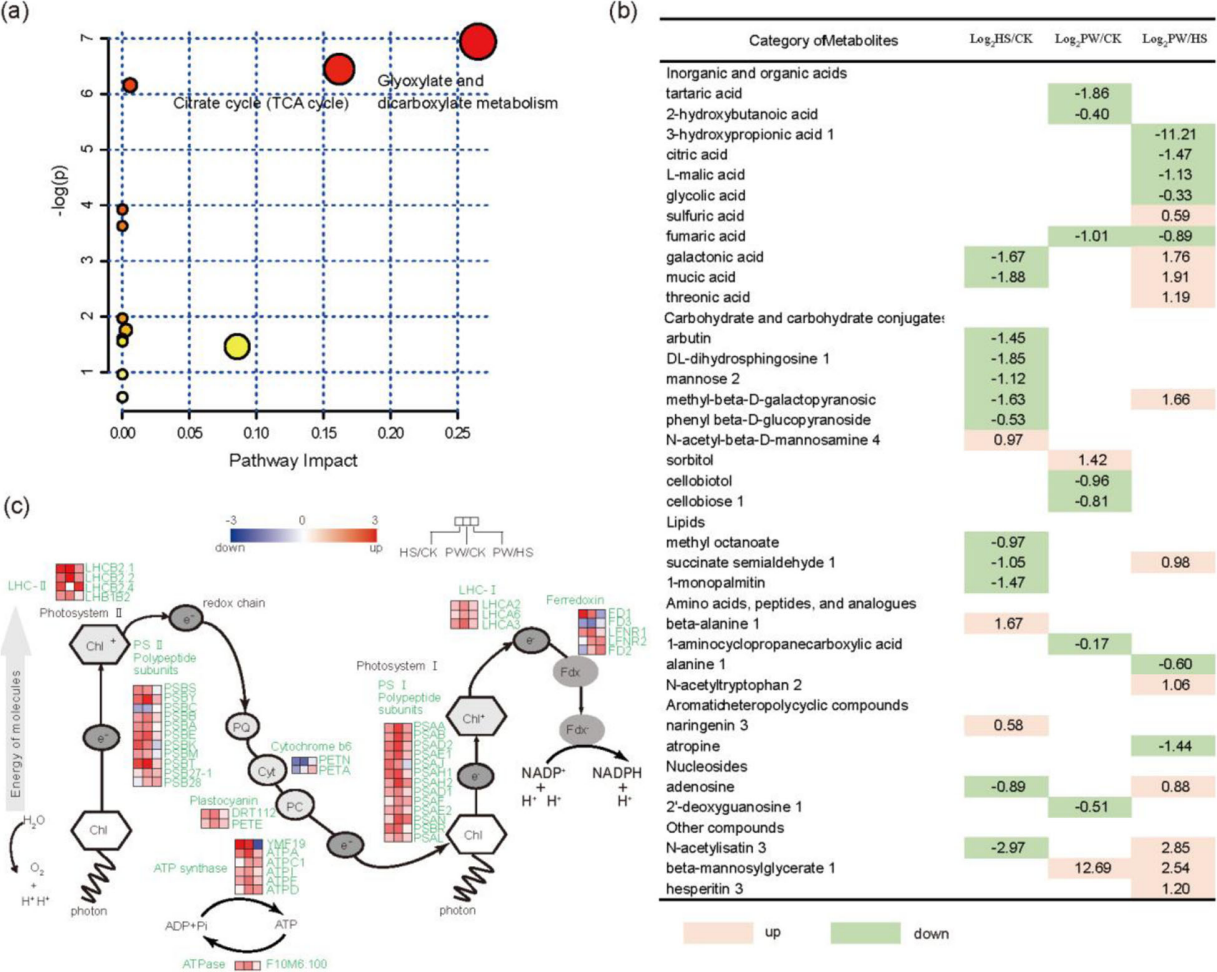
Metabolomic analyses and changes in the expression of photosynthesis-related genes of *A. thaliana* under different elevated temperature treatments. **a** Metabolome view map of the common metabolites identified in the plants subjected to prolonged warming and heat shock; the different colour depth of circles represent the *p*-value of pathway enrichment analysis. **b** List of metabolites significantly affected by heat shock (HS) compared to control (CK), prolonged warming (PW) compared to control (CK), prolonged warming (PW) compared to heat shock (HS) (*p*-value ≤0.05), organized by functional categories and corresponding accumulation fold-changes. The numbers represent fold-changes. Red shading means up-regulation and green shading means down-regulation**. c** Pathway diagram of light and dark reactions of photosynthesis with superimposed color-coded squares showing DEGs. Red squares: up-regulated genes; blue squares: down-regulated genes. Left-hand column: heat shock vs control; middle column: prolonged warming vs control; right-hand column: prolonged warming vs heat shock. CK: control; PW: prolonged warming; HS: heat shock. LHC I/II: light-harvesting complex I/II; PS I/II: photosystem I/II; PQ: plastoquinone; Cyt: cytochrome b6f complex; PC: plastocyanin; Chl: chlorophyll; Fdx: ferredoxin

Using GC-MS, we identified 181 metabolites that were significantly affected under prolonged warming and heat shock. Using principal component analysis (PCA) and orthogonal projection to latent structure with discriminant analysis (OPLS-DA), we separated these metabolites between experimental groups (Additional files 1: Figure S1a-d), and narrowed them down to 34 different metabolites (VIP > 1 and *p* < 0.05). The metabolome view map revealed that the enriched pathways (*p* < 0.05) between prolonged warming and heat shock were those involved in the citrate cycle, and glyoxylate and dicarboxylate metabolism (Fig. 2a).

The major metabolites differently accumulated among three treatments were listed in Fig. 2b. From the overall metabolite scenario, there was no common metabolites between heat shock vs CK and prolonged warming vs CK, indicating that these two types of heat stresses led to completely differential trends of metabolite changes. For example, sorbitol was significantly increased in prolonged warming, while it was not found in heat shock. Some carbohydrate conjugates (such as DL-dihydrosphingosine, mannose, methyl-beta-D-galactopyranosic, and phenyl-beta-D-glucopyranoside) were decreased in heat shock (compared to CK), while they showed no changes in prolonged warming (compared to CK). Furthermore, many metabolites involved in intermediates of the TCA cycle such as fumaric acid, L-malic acid and citric acid were significantly decreased in prolonged warming (PW vs HS) (Fig. 2b).

### Differentially expressed genes (DEGs) associated with photosynthesis

We generated RNA-Seq data from the leaves and obtained clean reads from three biological replicates each of CK (102,596,706), prolonged warming (77,761,052), and heat shock (80,456,340) treatment, respectively (Additional files 4: Table S1). We further carried out differential expression analysis (Additional files 2: Figure S2a). Based on KEGG (Kyoto Encyclopedia of Genes and Genomes) enrichment analysis, genes involved in ribosome, photosynthesis, antenna proteins, and citrate cycle were enriched in both prolonged warming and heat shock (Additional files 2: Figure S2b, c, red arrows). On the other hand, the pathways of porphyrin and chlorophyll metabolism and the biosynthesis of unsaturated fatty acids were significantly enriched in prolonged warming compared to heat shock (Additional files 2: Figure S2d, red arrows). Based on GO (gene ontology) enrichments analysis, heat shock resulted in the enrichment of genes associated response to stimulus, response to stress, cellular component and membrane (Additional files 3: Figure S3a, arrows), while heat shock resulted in the enrichment of genes associated with response to abiotic stimulus, chloroplast, plastid, cytoplasm, intracellular part, photosynthesis and light reaction (Additional files 3: Figure S3b, arrows). Between the two elevated temperature treatments (prolonged warming vs heat shock), genes associated with response to stimulus, response to stress, chloroplast, plastid, cytoplasm and cytoplasmic part were enriched (Additional files 3: Figure S3c, arrows).

Informed by our KEGG and GO enrichment results, we further investigated DEGs associated with the photosynthetic electron transport system. We identified four DEGs involved in light harvesting complex II (LHC II), three DEGs encoding light harvesting complex I (LHC I), 11 DEGs related to PS II and 13 DEGs related to PS I (Fig. 2c). Most of them were up-regulated in both prolonged warming and heat shock (except for *PSBC*, encoding the CP43 subunit of PS II). Particularly, *LHCB2.2* and *LHCB2.4* (encoding light harvesting complex II), *PSB28* (associated with PS II), and *PSAH2* and *PSAN* (related to PS I), were up-regulated by more than 2-fold in prolonged warming than in heat shock (PW vs HS) (Fig. 2c). In addition, ATP synthase (*ATPC1* and *ATPD*) in the photosynthetic electron transport system were also up-regulated in prolonged warming vs heat shock, while *YMF19* was down-regulated (Fig. 2c).

### Respiratory metabolism

**Fig. 3:**
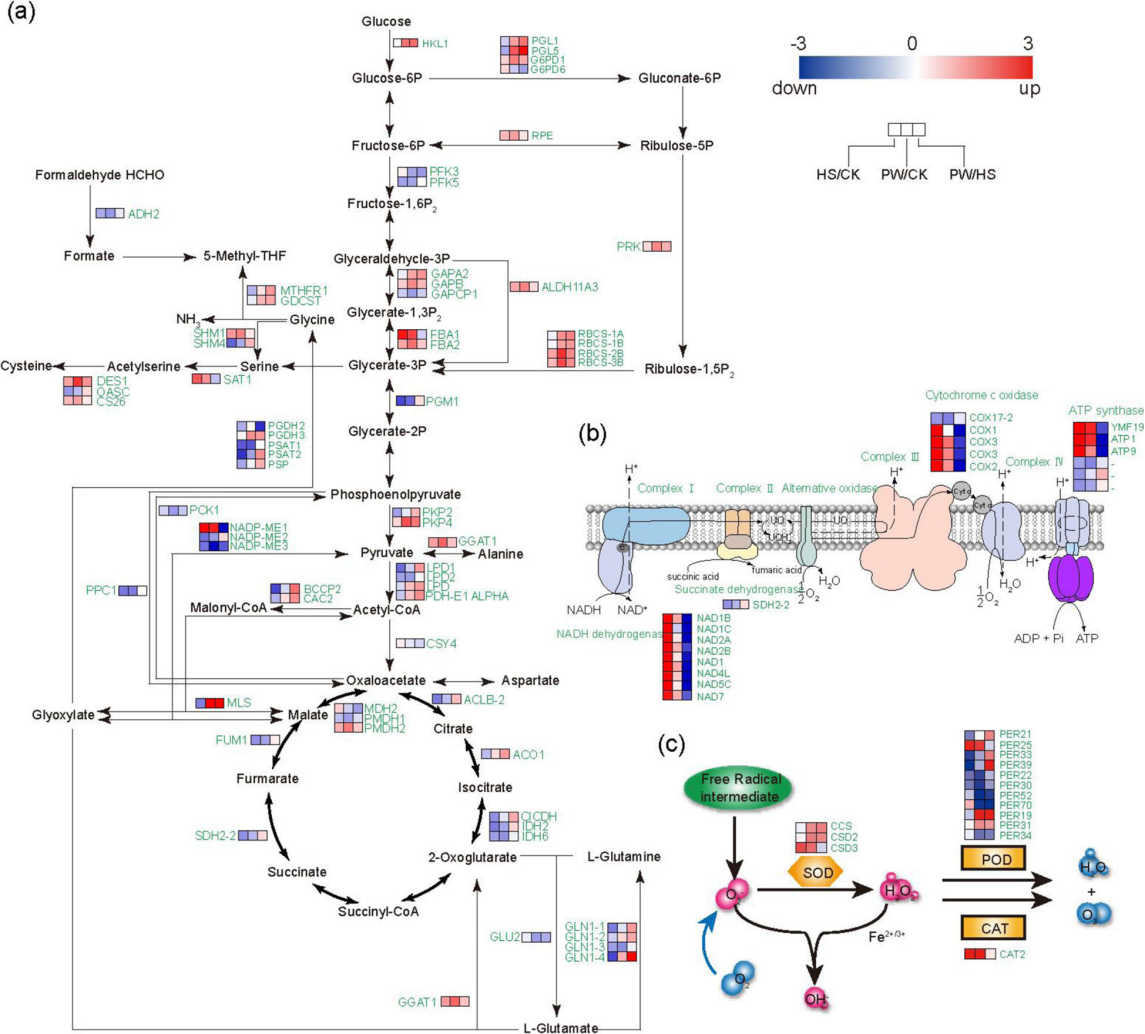
The respiratory metabolism and scavenging free radicals pathways analysis under different elevated temperature treatments. **a** The substance transformation and gene expression changes in the respiratory metabolism pathways under different elevated temperature treatments. **b** Changes in the expressions of oxidative phosphorylation-related genes in the mitochondrial electron transport chain. **c** Profiles of antioxidant enzyme-related genes responsible for scavenging free radicals. SOD: superoxide dismutase; CAT: catalase; POD: peroxidase. Red squares represent up-regulated genes and blue squares represent down-regulated ones. Left-hand column represents heat stress vs control; middle column represents warming vs control; right-hand column represents warming vs heat stress. CK: control; PW: prolonged warming; HS: heat shock

The expression levels of DEGs regulating glyoxylate and dicarboxylate metabolism dramatically increased in prolonged warming vs heat shock. For instance, the expression level of 40-fold higher *MLS* (encoding malate synthase), more than 8-fold higher *RBCS-1A*, *RBCS-1B*, *RBCS-2B* and *RBCS-3B* (encoding ribulose bisphosphate carboxylase), and 3-fold higher *HKL1* (encoding hexokinase-like 1), in prolonged warming than in heat shock (Fig. 3a).

By examining the relationship between the expression levels of DEGs and the abundance of metabolites, we identified those pathways that were significantly influenced by both elevated temperature treatments. The major known pathways, including glycolysis, pyruvate metabolism, glyoxylate and dicarboxylate metabolism and TCA cycle, are represented in Fig. 3a. Compared to CK, the metabolism of pyruvate genes encoding dihydrolipoyl dehydrogenase (*LPD1*, *LPD2*) were down-regulated in heat shock, indicated that heat stress limited the conversion of pyruvate into acetyl-CoA. However, *LPD1, LPD* and *PDH-E1 ALPHA* were up-regulated in prolonged warming vs heat shock, indicating a different pattern in this conversion between prolonged warming and heat shock.

In the TCA cycle pathway, genes encoding malate dehydrogenase (*PMDH1*), fumarate hydratase (*FUM1*), ATP-citrate synthase beta chain protein (*ACLB-2*), isocitrate dehydrogenase (*IDH2*, *IDH6*) and succinate dehydrogenase (*SDH2–2*) were down-regulated in both prolonged warming and heat shock. This is consistent with the metabolomic results, where citric acid and fumaric acid contents were decreased in prolonged warming vs heat shock (Fig. 2b, Fig. 3a). The decreased malic acid content was also consistent with the down-regulation of *PMDH1* in prolonged warming vs heat shock (Fig. 2b, Fig. 3a).

In addition, the DEGs related to oxidative phosphorylation, encoding ATP synthase, cytochrome c oxidase and NADH dehydrogenase, were up-regulated in HS. Examples are ATP synthase protein (*YMF19*), cytochrome c oxidase subunit (*COX1*, *COX2*, *COX3*), and NADH dehydrogenase (*NAD1B NAD1C*) (Fig. 3b). However, most these genes were significantly down-regulated in prolonged warming vs heat shock, indicated that heat shock enhanced more oxidative phosphorylation than prolonged warming.

### The antioxidant system

In SOD-catalyzed reactions, three genes (*CCS*, *CSD2*, *CSD3*) were all up-regulated in prolonged warming. However, only *CSD3* was highly expressed in heat shock. Additionally, the expression levels of *CCS* and *CSD2* were 2.5-fold higher in prolonged warming than in heat shock (Fig. 3c).

In CAT-catalyzed reactions, *CAT2* (catalase 2) had a higher expression in both heat shock and prolonged warming (Fig. 3c). In addition, most of the genes encoding PODs were down-regulated in heat shock or prolonged warming compared to CK, except for *PER25* (peroxidase 25) which had a higher expression in heat shock (Fig. 3c).

### Heat shock proteins, transcription factors and heat stress-inducible genes

**Fig. 4:**
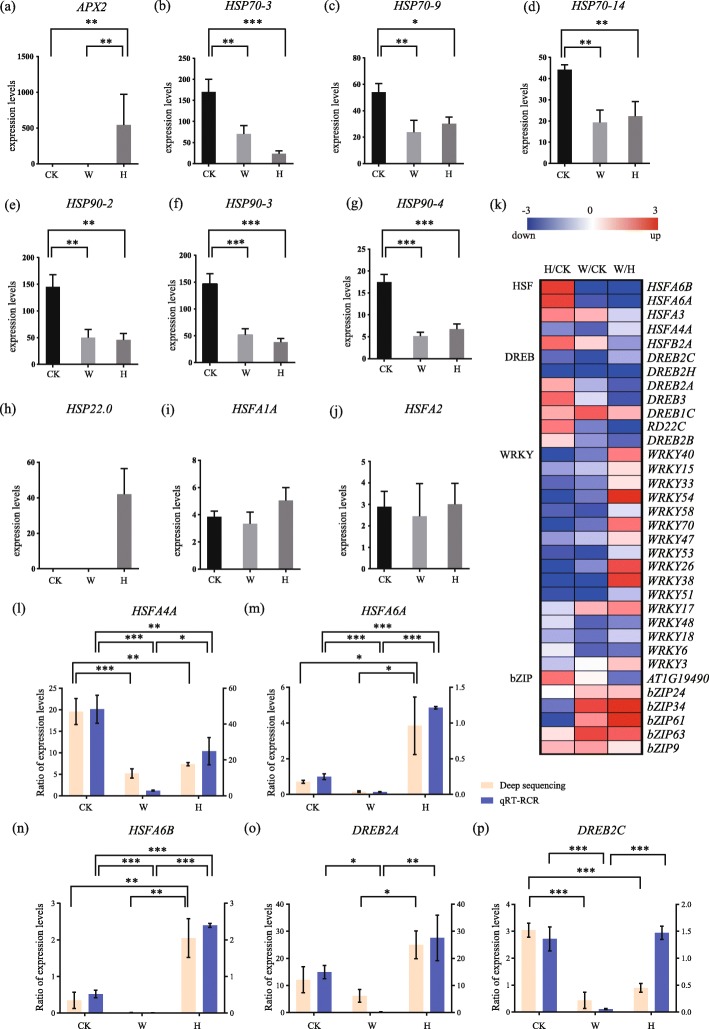
Heat shock protein and transcription factor analyses under different elevated temperature treatments. Expression levels of (**a**) ascorbate peroxidase 2 (*APX2*), (**b**) heat shock protein 70–3 (*HSP70–3*), (**c**) *HSP70–9*, (**d**) *HSP70–14*, (**e**) *HSP90–2*, (**f**) *HSP90–3*, (**g**) *HSP90–4*, (**h**) *HSP22.0*, (**i**) heat shock factor A 1A (*HSFA1A*), and **(j)** *HSFA2*. Each bar represents mean ± SD; *n* = 3. **k** Heatmap of 34 differentially expressed transcription factors under control, prolonged warming and heat shock treatments, grouped into 4 main categories. Red rectangles mean up-regulation of expression and blue means down-regulation. **l–p** The expression levels of several selected transcription factors: (**l**) *HSFA4A*, (**m**) *HSFA6A*, (**n**) *HSFA6B*, (**o**) *DREB2A*, and (**p**) *DREB2C* were determined using quantitative RT-PCR analyses. Pink bars: results from deep sequencing; blue bars: results from qRT-PCR. Each bar represents mean ± SD; *n* = 3. CK: control; PW: prolonged warming; HS: heat shock

The heat shock response network is activated by heat shock proteins thereby initiate heat-stress related transcription factors and genes. Ascorbate peroxidase 2 (*APX2*) is involved in catalyzing the H_2_O_2_-dependent oxidation of ascorbate in plants. We found that *APX2* were only significantly up-regulated in heat shock, but its expression was not detectable in prolonged warming (Fig. 4a). In addition, the expression levels of *HSP70–3*, *HSP70–9*, *HSP70–14*, *HSP90–2*, *HSP90–3* and *HSP90–4* were significantly decreased in both prolonged warming and heat shock, and the expression of *HSP70–3* was further decreased in heat shock than in prolonged warming (Fig. 4b-g). However, *HSP22.0* expression was only detected in heat shock, but not in prolonged warming (Fig. 4h).

By going a step upstream, we measured the expression levels of transcription factors including *HSFs*, *DREBs*, *WRKYs* and *bZIPs*. The expression levels of *HSFA1A* was slightly up-regulated in heat shock (Fig. 4i), but there was no significant difference in *HSFA2* expression among CK, prolonged warming and heat shock (Fig. 4j). Interestingly, four *HSFs*, including *HSFA6B, HSFA6A, HSFA3* and *HSFB2A* were significantly up-regulated, and *DREB2A* and *DREB3* were also strongly up-regulated in heat shock (Fig. 4k). Differently, the expressions of *DREB2A* and *DREB3* decreased in prolonged warming, and most genes encoding WRKY transcription factors were down-regulated to a larger extent in heat shock than those in prolonged warming (Fig. 4k). Particularly, five genes encoding bZIPs were significantly up-regulated in prolonged warming but only one (bZIP9) in heat shock (Fig. 4k). The expressions of *HSFA4A*, *HSFA6A*, *HSFA6B* and *DREB2A* detected via qRT-PCR (real-time reverse transcription PCR) showed a similar pattern to those observed in the transcriptome data (Fig. 4l-o), with DREB2C as an exception (Fig. 4p).

**Fig. 5:**
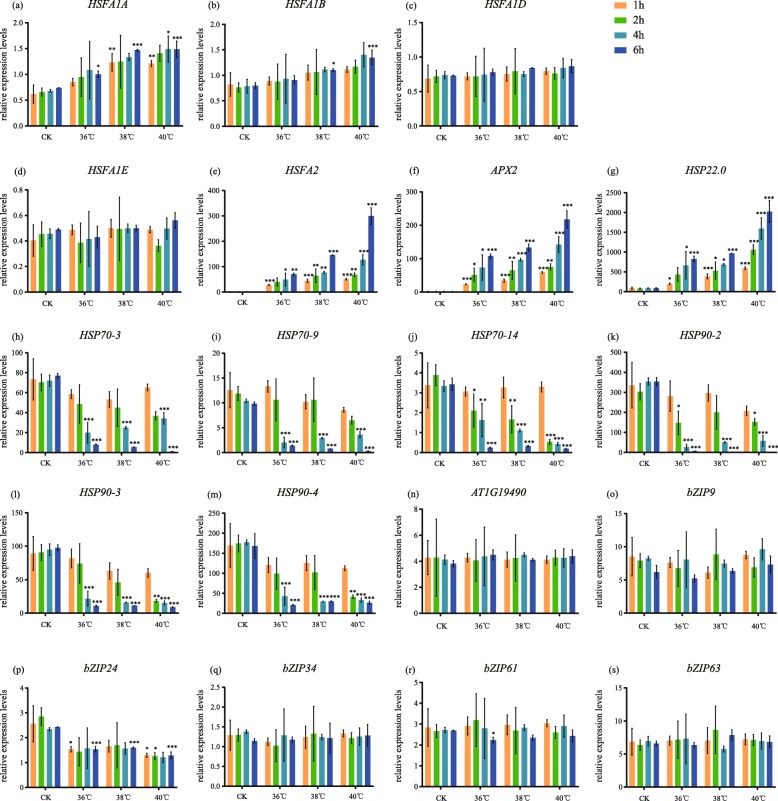
qRT-PCR for different temperatures and lasting time of heat shock. **a** *HSFA1A*, (**b**) *HSFA1B*, (**c**) *HSFA1D*, (**d**) *HSFA1E*, (**e**) *HSFA2*, (**f**) *APX2*, (**g**) *HSP22.0*, (**h**) *HSP70–3*, (**i**) *HSP70–9*, (**j**) *HSP70–14*, (**k**) *HSP90–2*, (**l**) *HSP90–3*, (**m**) *HSP90–4*, (**n**) *AT1G19490*, (**o**) *bZIP9*, (**p**) *bZIP24*, (**q**) *bZIP34*, (**r**) *bZIP61*, (**s**) *bZIP63*. Orange bars: results for 1 h, green bars: results for 2 h, blue bars: results for 4 h, deep blue bars: results for 6 h. Each bar represents mean ± SD; *n* = 3

**Fig. 6:**
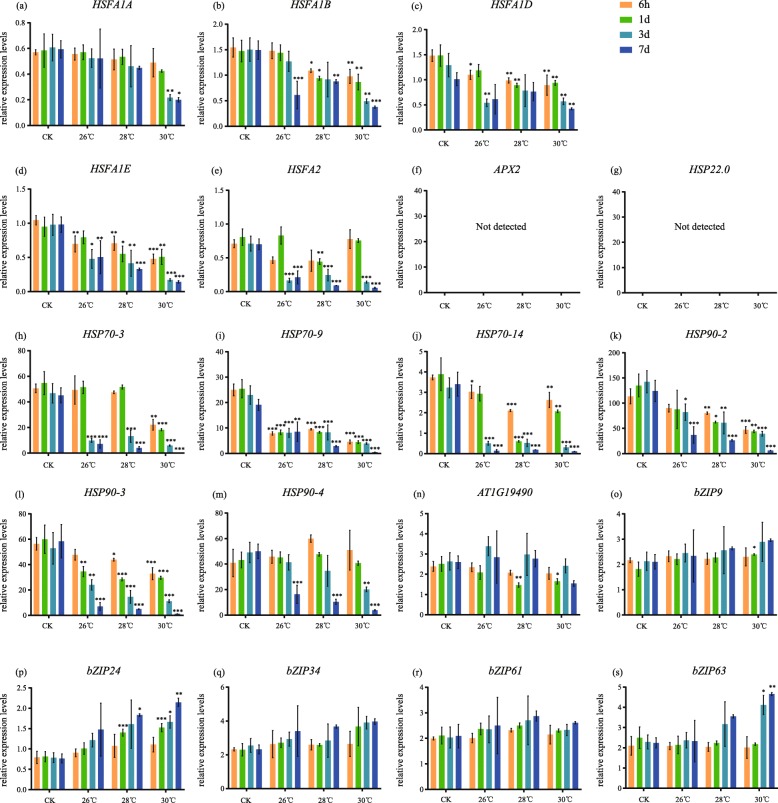
qRT-PCR for different temperatures and lasting time of prolonged warming. **a** *HSFA1A*, (**b**) *HSFA1B*, (**c**) *HSFA1D*, (**d**) *HSFA1E*, (**e**) *HSFA2*, (**f**) *APX2*, (**g**) *HSP22.0*, (**h**) *HSP70–3*, (**i**) *HSP70–9*, (**j**) *HSP70–14*, (**k**) *HSP90–2*, (**l**) *HSP90–3*, (**m**) *HSP90–4*, (**n**) *AT1G19490*, (**o**) *bZIP9*, (**p**) *bZIP24*, (**q**) *bZIP34*, (**r**) *bZIP61*, (**s**) *bZIP63*. Orange bars: results for 6 h, green bars: results for 1d, blue bars: results for 3d, deep blue bars: results for 7d. Each bar represents mean ± SD; *n* = 3

We further carried out a series of qRT-PCRs to verify the expression pattern of HSFAs and HSP70/90 in different temperatures with different durations (Fig. 5a-m, Fig. 6a-m, Additional files 5: Table S2). The results showed that in all heat treatments, the *HSFA1A*, *HSFA1B*, *HSFA2*, *APX2* and *HSP22.0* were significantly upregulated with the increasing of treatment duration (Fig. 5a, b, e, f, g), except that *HSFA1D* and *HSFA1E* showed little changes (Fig. 5c, d). However, unlike heat treatments, all *HSFA1s* and *HSFA2* were downregulated as the treated time increasing in all warming treatments (Fig. 6a-e) while *APX2* and *HSP22.0* were not detected (Fig. 6f, g), indicating that they were largely repressed under prolonged warming. In addition, *HSP70/90s* were all downregulated with the increasing treating time in both heat shock and prolonged warming (Fig. 5h-m, Fig. 6h-m). Among them, under heat shock, *HSPs*, *HSP70–3*, *HSP70–9*, *HSP70–14*, *HSP90–2*, *HSP90–3* and *HSP90–4*, exhibited the reverse expression level compared to the HSFA1s.

Most bZIPs were upregulated in prolonged warming on the 7th day, such as *bZIP9*, *bZIP24*, *bZIP34* and *bZIP63* (Fig. 6o, p, q, s). On the other hand, they showed no significantly changes under heat shock (Fig. 5o, p, q, s) except for the downregulated *bZIP24* (Fig. 5p), indicating that bZIPs were active in prolonged warming, which were totally different from their expressions in heat shock response.

## Discussion

By systematic comparison of the physiological, transcriptional, and metabolic responses of *Arabidopsis* leaves toward prolonged warming and heat shock, we revealed the differential response patterns between these two types of heat stresses, which involve multiple components including photosynthesis, respiration, ROS scavenging, and stress signaling pathways.

To determine the gas exchange under prolonged warming and heat shock conditions, we measured the stomatal conductance. We found that with prolonged warming treatment, the stomatal conductance decreased significantly, and the rate of CO_2_ assimilation was also significantly inhibited. However, they remained largely unchanged under heat shock which led to high transpiration rate. Stomatal regulation is a vital protective mechanism for high-temperature tolerance as it is crucial for desiccation prevention. Generally, under moderate to severe stress, the photosynthetic rate would decrease due to lowered mesophyll conductance and stomatal closure [12]. Therefore, our results suggest two different response mechanisms. Under heat shock, because plants experience a sudden increase in temperature, they may increase the rate of transpiration to allow the cooling effect due to evaporation. Differently, under prolonged warming in which the temperature increase is not lethal, the plants close their stomata to prevent excessive water loss.

We further investigated the responses of photosynthesis under prolonged warming and heat shock, and revealed that photochemical quenching decreased and non-photochemical quenching increased under prolonged warming, while the genes related to LHCII and the photosynthetic electron transport system including PSII and PSI, such as *LHCB2.2*, *LHCB2.4*, *PSB28*, *PSAH2*, *PSAN*, were up-regulated. Previous studies have identified that some PSII-related protein subunits and cofactors of the photosynthetic electron transport system responsive to high temperature. *Psb28–1* plays an important role in PSII repair at high temperatures [16, 17]. LHCII, as the major component of PSII, functions in light energy distribution and light protection, and Lhcb1 and Lhcb2 are primary constituents of mobile trimeric LHCIIs [18]. Combining with these studies, we predicted that the enhanced photoreaction and photoprotection under prolonged warming condition. Similarly, most genes related to light harvesting complexes and the photosynthetic electron transport system were also up-regulated with heat shock, suggesting that short-term high temperature may also induce photoprotection.

Respiration usually involves pathways of the glycolysis, TCA cycle, mitochondrial electron transport chain (miETC) and oxidative phosphorylation. Elevated temperatures can induce damages in the plant cell by upsetting the balance in cellular respiration [1]. Our data showed that the glycolysis pathway-related genes, including *HKL1*, *GAPA2*, *GAPB*, *FBA1*, *FBA2* and *PKP4*, were up-regulated with prolonged warming treatment. Meanwhile, the genes associated with the TCA cycle, such as *FUM1*, *PMDH1* and *ACLB-2*, were down-regulated, and the metabolomics results confirmed the decrease in TCA cycle activity. These results suggested that some specific TCA cycle intermediates were highly depleted by prolonged warming conditions. On the contrary, under heat shock, some glycolysis pathway-related genes, such as *PKP2*, *LPD1* and *LPD2* were down-regulated, while some genes related to the respiratory electron transfer and oxidative phosphorylation pathways, such as *NAD1B*, *NAD1C*, *COX1*, *COX2*, and *COX3*, were significantly induced, indicating that heat stress inhibited the glycolysis and TCA cycle pathways while enhancing electron transport.

Soluble carbohydrates and amino acids (such as proline) are important primary metabolites related to heat stress in plants, which were synthesized from the intermidiate metabolites from glycolysis and TCA cycle. The accumulation of soluble sugars, which are associated with cellular osmotic homeostasis and membrane stability, could protect the photosynthetic apparatus from heat damage and maintain photosynthetic capacity [19, 20]. In this study, soluble sugars were significantly increased under both prolonged warming and heat shock. Compared to prolonged warming, heat shock resulted in markedly higher concentrations of soluble sugars. In addition, the patterns of sugar alcohol and carbohydrate conjugate accumulation in response to the two treatments were quite different. For example, sorbitol accumulated extensively only with prolonged warming but not under heat shock, while galactonic acid, mannose, methyl-beta-D-galactopyranoside, and phenyl-beta-D-glucopyranoside were significantly reduced under heat shock. Since the osmotic substances were produced through photosynthetic assimilates or respiratory intermediate products, the ATP and NADPH were needed as the reducing power provider, which is mainly generated from respiration. Our results suggest that the molecules needed for maintaining osmotic balance during prolonged warming and heat shock might have been produced through the intermediate products of glycolysis.

Under abiotic stresses, plants usually accumulate ROS. At the same time, plants have expeditious antioxidant systems, including non-enzymatic antioxidants such as ascorbate (ASC) and glutathione (GSH), as well as antioxidant enzymes such as SOD, POD and CAT responsible for ROS scavenging and removal. However, once the equilibrium between the generation and scavenging of ROS is disrupted under stress conditions, ROS start to accumulate [21, 22]. Here we found that the concentration of H_2_O_2_, the activities of SOD and CAT, and their related genes, including *CSD3* and *CAT2*, were all enhanced under both prolonged warming and heat shock treatments, indicating that both types of treatments can induce ROS-scavenging enzyme activities to detoxify ROS. However, the concentration of H_2_O_2_ increased more dramatically upon rapid heat shock. Moreover, POD activity was higher under heat shock than with prolonged warming, and POD synthesis-related gene *PER25* expression was significantly up-regulated under heat shock vs prolonged warming, indicating the significant accumulation of ROS, and the disruption of the equilibrium between ROS generation and scavenging systems under heat shock. With prolonged warming, the level of ROS, although elevated from the control level, was still relatively low. Actually, several lines of evidence have shown that when under moderate stress, the scavenging system could keep the ROS level low, with ROS serving as signaling molecules which activate an acclimation response and programmed cell death. For example, at moderate stress, ROS play a crucial role in intracellular signaling from the chloroplast to the nucleus to control plant development processes [23]. Therefore, at this point, we postulate that ROS might function as signaling molecules to regulate the activation of stress response pathways, and did not result in the irreversible inactivation of the photosynthetic system or cause grave damage to PSII under prolonged warming. However, the detailed mechanisms await further investigation.

In anticipation of upcoming damaging conditions, plants can activate genes and accumulate HSPs involved in cellular defenses against heat damage. HSPs, including HSP100, HSP90, HSP70, HSP60, and small HSPs, have critical roles in regulating protein quality by renaturing a variety of proteins denatured due to heat stress. These HSPs are in turn precisely controlled by a network of transcription factors (TFs), including HSFs, DREBs, WRKYs, and bZIPs [24]. Recent reviews have elucidated the complex transcriptional and post-translational regulatory networks involved in heat stress [13, 25]. HSFs are the terminal components of a signal transduction chain mediating the activation of genes responsive to heat stress, which are particularly important in thermotolerance responses [13]. In this study, transcriptome analysis by RNA-seq detected 33 TF families, including HSFs, DREBs, WRKYs, and bZIPs that responded to heat stress. Among them, the transcription factors in HSFA1s and DREBs pathways, such as *HSFA1A*, *HSFA6A*, *HSFA6B* and *DREB2A* were up-regulated in heat shock treatment. In *Arabidopsis*, HSFA1s were shown to play a central role in the heat stress response. Many important heat stress response TFs, such as DREB2A, HSFA2, HSFA7a, and HSFBs have been predicted to be directly regulated by HSFA1s [26]. Therefore, our data suggest that the HSFA1s and DREBs play a crucial role in response to heat shock. HSP70 and HSP90 can repress HSFA1 activity through the repression of its transactivation activity and nuclear localization, respectively. Upon heat shock, HSFA1s are dissociated from HSP70 and de-repressed [13]. Similarly, our qRT-PCR results showed that all *HSP70/90s* were significantly down-regulated and their corresponding *HSFA1s* were up-regulated in heat shock. These results further confirmed that HSFA1 becomes active from the repression of HSP70/90, and HSFA1s are negatively regulated by HSP70/90. However, in prolonged warming treatments, although the *HSP70/90s* showed low expression levels, the *HSFA1s* were downregulated, suggesting that HSFA1s did not act as the central regulator in response to prolonged warming.

bZIP TFs are endoplasmic reticulum stress sensors in plants, which regulate many processes including abscisic acid (ABA) and stress signaling, and contribute to stress tolerance [27]. In our study, it is interesting to point out that, with prolonged warming, the transcription factors in an HSFA1-independent pathway, such as *bZIP9*, *bZIP24*, *bZIP34* and *bZIP63* were up-regulated. In addition, our large scale qRT-PCR results also validated that these bZIP family members, such as *bZIP24* and *bZIP34*, were up-regulated in all prolonged warming treatments (e.g., the 7th day), whereas none of them were upregulated in heat shock. These results suggest that prolonged warming and heat shock may induce entirely different heat response pathways for thermo-tolerance or thermo-acclimation.

Besides, early exposure to a mild temperature stress can enhance thermotolerance to heat stress, and the stress priming may occur at the level of gene transcription, such as *HSFA2* expression depending on the expressed *HSFA1* isoforms [28, 29]. Additionally, *HSP22.0* and *APX2* associated with heat stress priming can remain elevated levels for several days in heat memory [28]. Here, we found that *HSFA2*, *HSP22.0* and *APX2* were only up-regulated under heat shock but not with prolonged warming, indicating that prolonged warming treatment conditions (5 °C above control) may not be enough to prime the plant to subsequently withstand high temperatures in *Arabidopsis*.

## Conclusions

**Fig. 7:**
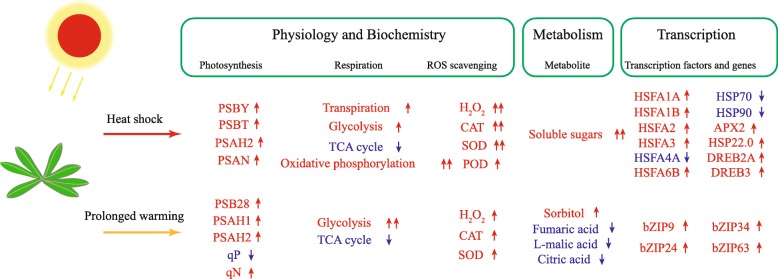
A schematic diagram summarizing the responses to prolonged warming and heat shock in *A. thaliana*. The changes of main indexes in physiology, biochemistry, metabolism and transcription are listed. Red represent up-regulation and blue represent down-regulation

Our research provided detailed information on the physiological, transcriptional, and metabolic responses of *Arabidopsis* to prolonged warming versus heat shock (summarized in Fig. 7). On the basis of these multi-level results, we conclude that plants respond to rapid-onset heat shock mainly through increasing the rate of transpiration, the rate of photosynthetic and respiratory electron transfer, the production of ROS, the induction of antioxidant enzymes, and the activation of the HSFA1 heat stress response pathway. On the other hand, plants respond to prolonged warming primarily via decreased stomatal conductance, increased photosynthetic electron transfer rate, inhibited TCA cycle, and activation of a HSFA1-independent response pathway of bZIPs.

## Methods

### Plant materials and growth conditions

The seeds of wild-type (WT) *Arabidopsis thaliana* Columbia ecotype (Col-0) were originally acquired from Nottingham *Arabidopsis* Stock Centre, Nottingham University, UK, and have grown in growth chambers at 23/18 °C (day/night) for more than 30 generations by seed propagation over the past 10 years in our laboratory. Seeds were stratified at 4 °C for 2 days, and then sown in pots (650 × 650 × 750 mm) filled with a mixture of vermiculite and peat (1:1, v/v), and placed in growth chambers (RXZ-300B, NingboDongnan Instruments Co Ltd., China) under 23 °C (16 h)/18 °C (8 h) (day/night) with day light intensity 350 μmol·m^− 2^·s^− 1^. Relative humidity (RH) was maintained at 80%/90% (day/night). After true leaf emergence, seedlings were thinned to two or three plants per pot and the pots were randomly rearranged every 3 days to offset position effects within the chambers (Fig. 1a). The plants were alternately watered with 1/2 Murashige and Skoog solution or with de-ionized water once a week. To eliminate possible differences among the different growth chambers, plants of each treatment were exchanged and relocated in different growth chambers (≥3) irregularly during plant growth and treatment. Besides, although the sensitivity of the growth chamber is in the range of ±0.5 °C, two thermometers were placed in each growth chamber for checking the accuracy of the setting temperatures.

#### Control

The day/night temperatures were set at 23/18 °C as the control temperature (CK) based on published studies using the Col-0 ecotype [8, 30]. Leaves were sampled from plants at 30 days after sowing (at the rosette growth stage) (Fig. 1b).

#### Prolonged warming treatment

The global mean temperature is likely to warm by 1.5–4 °C at the end of this century [31]. Therefore, we set the warming temperature at 5 °C above CK, and plants at 23 days after sowing were subjected to 28/23 °C (day/night) for 7 days as the prolonged warming treatment. After warming treatment, leaves were sampled for analyses (Fig. 1b).

#### Heat shock treatment

Since 37–42 °C (44–45 °C being the lethal temperature) had been widely used as the temperature in *Arabidopsis* heat stress studies, we set 38 °C for 6 h during the day portion of the photoperiod as the heat shock treatment (Fig. 1b).

Hence, all leaf sampling in the three temperature regimes were done with plants at 30 days after sowing (growth stage 3.90). At this stage, the plant rosette growth is nearly complete and leaves are fully expanded [32]. All leaf samples were immediately snap-frozen in liquid nitrogen. The same batch of sampling materials were used for transcriptome, metabolome, physiological and biochemical analyses.

### RNA extraction, RNA sequencing and data analyses

Total RNA was isolated separately from the leaves subjected to control, prolonged warming and heat shock treatments, respectively, with three biological replicates each, for RNA extraction and sequencing. All total RNA samples were extracted using the Mini BEST Plant RNA Extraction Kit (TaKaRa, Dalian, China) and treated with genomic DNA Eraser (TaKaRa, Dalian, China) to reduce or eliminate any DNA contamination. Illumina-based RNA sequencing was performed on the Hiseq™ 4000 platform. After removing the reads containing adapter, reads containing ploy-N and low quality reads, the filtered reads were mapped to the *A. thaliana* genome (TAIR 10) using TopHat2. Then Reads Per Kilobase of transcript per Million mapped reads (RPKM) of each gene was calculated based on the length of the gene and reads count mapped to this gene. Differential expression analysis among the samples was performed using the DESeq R package (1.18.0). Thresholds of |log_2_(fold change)| ≥ 1 and adjusted *P*-value < 0.05 were applied to assess the significance of the differences in transcript levels. The sequencing data have been deposited in the NCBI Gene Expression Omnibus (GEO) database under the accession number GSE118298.

### Metabolome analysis

A volume of 0.48 mL methanol–water (3:1, v/v) and 24 μL of adonitol (1 mg/mL stock in dH_2_O) were added to 0.06 g of each sample in a 2 mL Eppendorf tube as internal standard, followed by homogenization in a ball mill for 4 min at 50 Hz, and then sonication for 5 min twice (with incubation in ice water). After centrifugation at 13000×g at 4 °C for 15 min, 350 μL of supernatant was transferred into a fresh 2 mL GC/MS glass vial. After drying the samples with a vacuum concentrator, 80 μL methoxyamine hydrochloride (20 mg/mL in pyridine) was added to each sample and incubated at 80 °C for 30 min. Then, 100 μL of the BSTFA regent (1% TMCS, v/v) was added and the mixture incubated at 70 °C for 1.5 h and mixed well for GC–time-of-flight (TOF)–MS analysis.

GC-TOF-MS analysis was performed using an Agilent 7890 gas chromatograph system coupled with a Pegasus HT time-of-flight mass spectrometer (Agilent Technologies, Santa Clara, CA, USA). Each treatment in this metabolomics study was repeated with at least six biological replicates.

Chroma TOF 4.3X software of LECO Corporation and LECO-Fiehn Rtx5 database were used for raw peaks exacting, data baselines filtering, baseline calibration, peak alignment, deconvolution analysis, peak identification and integration of the peak area. The RI (retention time index) method was used for peak identification, with the RI tolerance at 5000.

### Measurement of photosynthetic capacity

The main photosynthetic parameters were measured on mature leaves using a Portable Photosynthesis System (LI-6400XT) to quantify CO_2_ uptake under conditions of saturating light and water availability. All samples were measured on intact plants in the growth chambers under the three different treatments. During all measurements, a high flow rate (400 mL·min^− 1^) through the cuvette was maintained to keep the CO_2_ concentration within the range of 370–390 μmol·mol^− 1^. The temperature in the leaf chamber was kept the same as the treatment temperature and all measurements were carried out between the eighth and ninth hour of daylight in the growth chambers. Light intensities of 1000 μmol quanta m^− 2^·s^− 1^ were used in the Photosynthesis System as the saturating photosynthetic photon flux density of *Arabidopsis*. Eight leaves from eight different plants were measured to provide biological replicates in each treatment. All the data collected at steady-state after inserting leaves into the leaf chamber.

### H_2_O_2_ level analysis

Freshly cut leaf samples (0.1 g) were homogenized in an ice bath with 0.9 mL of 50 mmol·L^− 1^ phosphate buffer (PH7.8) and centrifuged at 10,000×g for 10 min at 4 °C. The H_2_O_2_ concentration in the supernatant was determined by a colorimetric method using a commercial kit (BCA assay, Nanjing Jiancheng Bioengineering Institute, Nanjing, China). Three to five leaves were used to provide enough amount of leaf tissues for each sample (three biological replicates per treatment).

### Physiological Indicator measurements

The soluble sugar concentration in the supernatant was determined by anthrone colorimetry, and the soluble protein concentration in the supernatant was determined by the Coomassie Brilliant Blue method [33]. Freshly cut leaf samples (0.1 g) were homogenized in an ice bath with 1 mL of distilled water and put into a water bath at 95 °C for 10 min. After cooling, the samples were centrifuged at 8000×g for 10 min at 25 °C, and diluted with distilled water to 10 mL. Three to five leaves were used to provide enough amounts of leaf tissues for each replicate (three biological replicates per treatment).

The superoxide radical scavenging ability in the supernatant was determined using a commercial kit (BCA assay, Nanjing Jiancheng Bioengineering Institute, Nanjing, China). The activities of three enzymes SOD, CAT and POD were determined using commercial kits (Suzhou Comin Bioengineering, Suzhou, China). Three to five leaves were used to provide enough amounts of leaf tissues for each replicate (three biological replicates per treatment).

### Real-time reverse transcription PCR (qRT-PCR) analysis

We performed qRT-PCR to verify the results of transcriptome. In addition, to validate the expression pattern of some selected genes encoding important functions such as transcription factors, we performed a series of qRT-PCRs in different temperatures with different durations. For heat shock, we included treatments at 36 °C, 38 °C and 40 °C, and sampled the leaves at 1 h, 2 h, 3 h and 6 h after treatments. For prolonged warming, we included treatments at 26 °C, 28 °C and 30 °C and sampled the leaves at 6 h, 1d, 3d and 7d after treatments. All leaf samples were immediately snap-frozen in liquid nitrogen.

Each RNA sample (containing about 1 μg of total RNA) was treated with gDNA Eraser (TaKaRa, Dalian, China) following the manufacturer’s instructions, to eliminate any contaminant gDNA. The treated RNA solution (10 μL) was subjected to reverse transcriptase reactions with PrimeScript™ Reverse Transcriptase Reagent Kit with gDNA Eraser (Perfect Real Time) (TaKaRa, Dalian, China) in accordance with the manufacturer’s protocol. Gene-specific primers were designed using Primer 5.0. *Actin2* mRNA was used as the internal reference gene. Quantitative RT-PCR was performed using a Bio-Rad CFX96™ Real-Time System (Bio-Rad, USA) using the SYBR Premix Ex Taq™ Kit (Perfect Real Time) (TaKaRa, Japan) in accordance with the manufacturer’s protocol. qRT-PCR conditions were as follows: 30 s at 94 °C for denaturation, 40 cycles for 5 s at 94 °C, 30 s at 56 °C, and 10 s at 72 °C. Relative expression levels of target genes were calculated with the 2^-△△Ct^ comparative threshold cycle (Ct) method. All reactions were performed in three biological replicates, and the results of Ct values were determined with Bio-Rad CFX Manager V1.6.541.1028 software.

### Statistical analysis

Statistical significance of differences in this study was analyzed using one-way ANOVA followed by Tukey’s post hoc test with a significance level of 0.05 (*p* < 0.05) (SPSS 18.0 software for Windows) (SPSS, Chicago, IL, USA) [34].

## Supplementary information

**Additional files 1: Figure S1.** Principal component analysis (PCA) and Orthogonal projection to latent structure with discriminant analysis (OPLS-DA) of Metabolomic analyses. **(a)** PCA scores plot. **(b)-(d)** OPLS-DA scores: **(b)** control (CK) vs heat shock (HS); **(c)** control (CK) vs prolonged warming (PW); **(d)** prolonged warming (PW) vs heat shock (HS). Black circle: control; red square: prolonged warming; blue triangle: heat shock. Each treatment contains six biological repeats.

**Additional file 2: Figure S2.** Gene expression comparisons. **(a)** Venn diagram of the number of differentially expressed genes (DEGs). Numbers in the overlapping sets show the number of genes that are differentially expression in two or three pairwise comparisons. **(b)-(d)** Kyoto Encyclopedia of Genes and Genomes (KEGG) pathway enrichment of DEGs. **(b)** Control (CK) vs heat shock (HS); **(c)** control (CK) vs prolonged warming (PW); **(d)** prolonged warming (PW) vs heat shock (HS). The sizes of the dots are in proportion to the number of genes involved. The color of the dot is closer to red as the q-value approaches 0. The genes are considered statisticallysignificantly over-represented, i.e. enriched, when q < 0.05. Red arrows in (b) and (c) indicate those pathway enrichment of DEGs involved in ribosome, antenna proteins, photosynthesis and citrate cycle; Red arrows in (d) indicate the most siginificant enriched pathways.

**Additional file 3: Figure S3.** Gene ontology (GO) term enrichment of DEGs. (a) Heat shock (HS) vs control (CK); (b) prolonged warming (PW) vs control (CK); (c) prolonged warming (PW) vs heat shock (HS) Green bars represent genes involved in biological processes in response to external stimuli, and orange bars represent those involved in the structures of cellular components.

**Additional file 4: Table S1**. Summary of draft reads of samples by Illumina deep sequencing.

**Additional file 5: Table S2.** Primers used in qRT-PCR of the leaves of *A. thaliana.*

## Data Availability

The sequencing data are available in the NCBI Gene Expression Omnibus (GEO) database under the accession number GSE118298. The datasets supporting the results of this article are included within the article and the additional files.

## Abbreviations

ABA: Abscisic acid
ACLB: ATP-citrate synthase beta chain
APX2: Ascorbate peroxidase 2
ASC: Ascorbate
bZIP: Basic leucine zipper
CAT: Catalase
CAT2: Catalase 2
CCS: Copper chaperone for superoxide dismutase
Chl: Chlorophyll
CK: Control
COX 1: Cytochrome c oxidase subunit 1
CSD: Superoxide dismutase
Cyt: Cytochrome b6f complex
DEGs: Differentially expressed genes
DREBs: Dehydration responsive element-binding protein
FBA1/2: Fructose-bisphosphate aldolase 1/2
Fdx: Ferredoxin
FUM1: Fumarate hydratase
GAPA2: Glyceraldehyde 3-phosphate dehydrogenase A subunit 2
GAPB: Glyceraldehyde-3-phosphate dehydrogenase B subunit
GEO: Gene Expression Omnibus
GO: Gene ontology
GSH: Glutathione
H_2_O_2_: Hydrogen peroxide
HKL 1: Hexokinase-like 1
HS: Heat shock
HSF: Heat shock factor
HSFA1: Class A1 heat shock factor
HSPs: Heat shock proteins
IDH: Isocitrate dehydrogenase
KEGG: Kyoto Encyclopedia of Genes and Genomes
LHC I/II: Light-harvesting complex I/II
LPD: Dihydrolipoyl dehydrogenase
miETC: Mitochondrial electron transport chain
MLS: Malate synthase
NAD1B/C: NADH dehydrogenase
NADH: Nicotinamide adenine dinucleotide
OPLS-DA: Orthogonal projection to latent structure with discriminant analysis
PC: Plastocyanin
PCA: Principal component analysis
PER25: Peroxidase 25
PKP4: Plastidial pyruvate kinase 4
PMDH1: Protein malate dehydrogenase 1
POD: Peroxidase
PQ: Plastoquinone
PS I/II: Photosystem I/II
PW: Prolonged warming
qN: Non-photochemical quenching
qP: Photochemical quenching
qRT-PCR: Real-time reverse transcription PCR
RBCS: Ribulose bisphosphate carboxylase
ROS: Reactive oxygen species
SDH: Succinate dehydrogenase
SEM: Scanning electron microscopy
SOD: Superoxide dismutase
TCA: The tricarboxylic acid
TFs: Transcription factors
WRKYs: WRKY transcription factors
YMF19: ATP synthase protein YMF19

## References

[1] Wahid A, Gelani S, Ashraf M, Foolad MR (2007). Heat tolerance in plants: an overview. Environ Exp Bot.

[2] Wolkovich EM, Cook BI, Allen JM, Crimmins TM, Betancourt JL, Travers SE (2012). Warming experiments underpredict plant phenological responses to climate change. Nature..

[3] Springate DA, Kover PX (2014). Plant responses to elevated temperatures: a field study on phenological sensitivity and fitness responses to simulated climate warming. Glob Chang Biol.

[4] Hedhly A, Hormaza JI, Herrero M (2009). Global warming and sexual plant reproduction. Trends Plant Sci.

[5] Ainsworth EA, Ort DR (2010). How do we improve crop production in a warming world?. Plant Physiol.

[6] Lin D, Xia J, Wan S (2010). Climate warming and biomass accumulation of terrestrial plants: a meta-analysis. New Phytol.

[7] Walther GR (2010). Community and ecosystem responses to recent climate change. Philos T R Soc B.

[8] Jin B, Li W, Jing W, Jiang KZ, Yang W, Jiang XX, Ni CY, Wang YL, Teng NJ (2011). The effect of experimental warming on leaf functional traits, leaf structure and leaf biochemistry in *Arabidopsis thaliana*. BMC Plant Biol.

[9] Way DA, Yamori W (2014). Thermal acclimation of photosynthesis: on the importance of adjusting our definitions and accounting for thermal acclimation of respiration. Photosynth Res.

[10] Glaubitz U, Li X, Schaedel S, Erban A, Sulpice R, Kopka J, Hincha DK, Zuther E (2017). Integrated analysis of rice transcriptomic and metabolomic responses to elevated night temperatures identifies sensitivity- and tolerance-related profiles. Plant Cell Environ.

[11] Pospíšil P (2016). Production of reactive oxygen species by photosystem II as a response to light and temperature stress. Front Plant Sci.

[12] Bita CE, Gerats T (2013). Plant tolerance to high temperature in a changing environment: scientific fundamentals and production of heat stress-tolerant crops. Front Plant Sci.

[13] Ohama N, Sato H, Shinozaki K, Yamaguchishinozaki K (2017). Transcriptional regulatory network of plant heat stress response. Trends Plant Sci.

[14] Niu Y, Xiang Y (2018). An overview of biomembrane functions in plant responses to high-temperature stress. Front Plant Sci.

[15] Atkin OK, Loveys BR, Atkinson LJ, Pons TL (2006). Phenotypic plasticity and growth temperature: understanding interspecific variability. J Exp Bot.

[16] Ashraf M, Harris PJC (2013). Photosynthesis under stressful environments: an overview. Photosynthetica.

[17] Sakata S, Mizusawa N, Kubotakawai H, Sakurai I, Wada H (2013). Psb28 is involved in recovery of photosystem II at high temperature in *Synechocystis* sp. PCC 6803. Biochim Biophys Acta.

[18] Allakhverdiev SI, Kreslavski VD, Klimov VV, Los DA, Carpentier R, Mohanty P (2008). Heat stress: an overview of molecular responses in photosynthesis. Photosynth Res.

[19] Patrick JW, Botha FC, Birch RG (2013). Metabolic engineering of sugars and simple sugar derivatives in plants. Plant Biotechnol J.

[20] Dumschott K, Richter A, Loescher W, Merchant A (2017). Post photosynthetic carbon partitioning to sugar alcohols and consequences for plant growth. Phytochemistry.

[21] Lismont C, Nordgren M, Van Veldhoven PP, Fransen M (2015). Redox interplay between mitochondria and peroxisomes. Front Cell Dev Biol.

[22] Dietz KJ, Turkan I, Krieger-Liszkay A (2016). Redox- and reactive oxygen species-dependent signaling into and out of the photosynthesizing chloroplast. Plant Physiol.

[23] Sun AZ, Guo FQ (2016). Chloroplast retrograde regulation of heat stress responses in plants. Front Plant Sci.

[24] Grover A, Mittal D, Negi M, Lavania D (2013). Generating high temperature tolerant transgenic plants: achievements and challenges. Plant Sci.

[25] Zhao J, He Q, Chen G, Wang L, Jin B (2016). Regulation of non-coding RNAs in heat stress responses of plants. Front Plant Sci.

[26] Yoshida T, Ohama N, Nakajima J, Kidokoro S, Mizoi J, Nakashima K (2011). *Arabidopsis* HSFA1 transcription factors function as the main positive regulators in heat shock-responsive gene expression. Mol Gen Genomics.

[27] Srivastava R, Deng Y, Howell SH (2014). Stress sensing in plants by an ER stress sensor/transducer, bZIP28. Front Plant Sci.

[28] Bäurle I (2016). Plant heat adaptation: priming in response to heat stress. F1000research.

[29] Lämke J, Brzezinka K, Altmann S, Bäurle I (2016). A hit- and -run heat shock factor governs sustained histone methylation and transcriptional stress memory. EMBO J.

[30] Balasubramanian S, Sureshkumar S, Lempe J, Weigel D (2006). Potent induction of *Arabidopsis thaliana* flowering by elevated growth temperature. PLoS Genet.

[31] Flato G, Marotzke J, Abiodun B, Braconnot P, Chou SC, Collins WJ, Stocker TF, Qin D, Plattner G-K, Tignor M, Allen SK, Boschung J, Nauels A, Xia Y, Bex V, Midgley PM (2013). Evaluation of climate models. Climate Change. 2013: The physical science basis. contribution of working group I to the fifth assessment report of the intergovernmental panel on climate change.

[32] Boyes DC, Zayed AM, Ascenzi R, Mccaskill AJ, Hoffman NE, Davis KR, Görlach J (2001). Growth stage-based phenotypic analysis of *Arabidopsis*: a model for high throughput functional genomics in plants. Plant Cell.

[33] Ren S, Ma K, Lu Z, Chen G, Cui J, Tong P, Wang L, Teng N, Jin B (2019). Transcriptomic and metabolomic analysis of the heat-stress response of *Populus tomentosa* Carr. Forests.

[34] Allen P, Bennett K, King J. PASW statistics by SPSS, a practical guide: version 18.0. Melbourne: Cengage Learning Press; 2010.

