# Supplementary figures and images for: Differential physiological, transcriptomic and metabolomic responses of Arabidopsis leaves under prolonged warming and heat shock
Source: BMC Plant Biol. 2020 Feb 22;20:86. doi: 10.1186/s12870-020-2292-y (PMC7036190; doi:10.1186/s12870-020-2292-y)

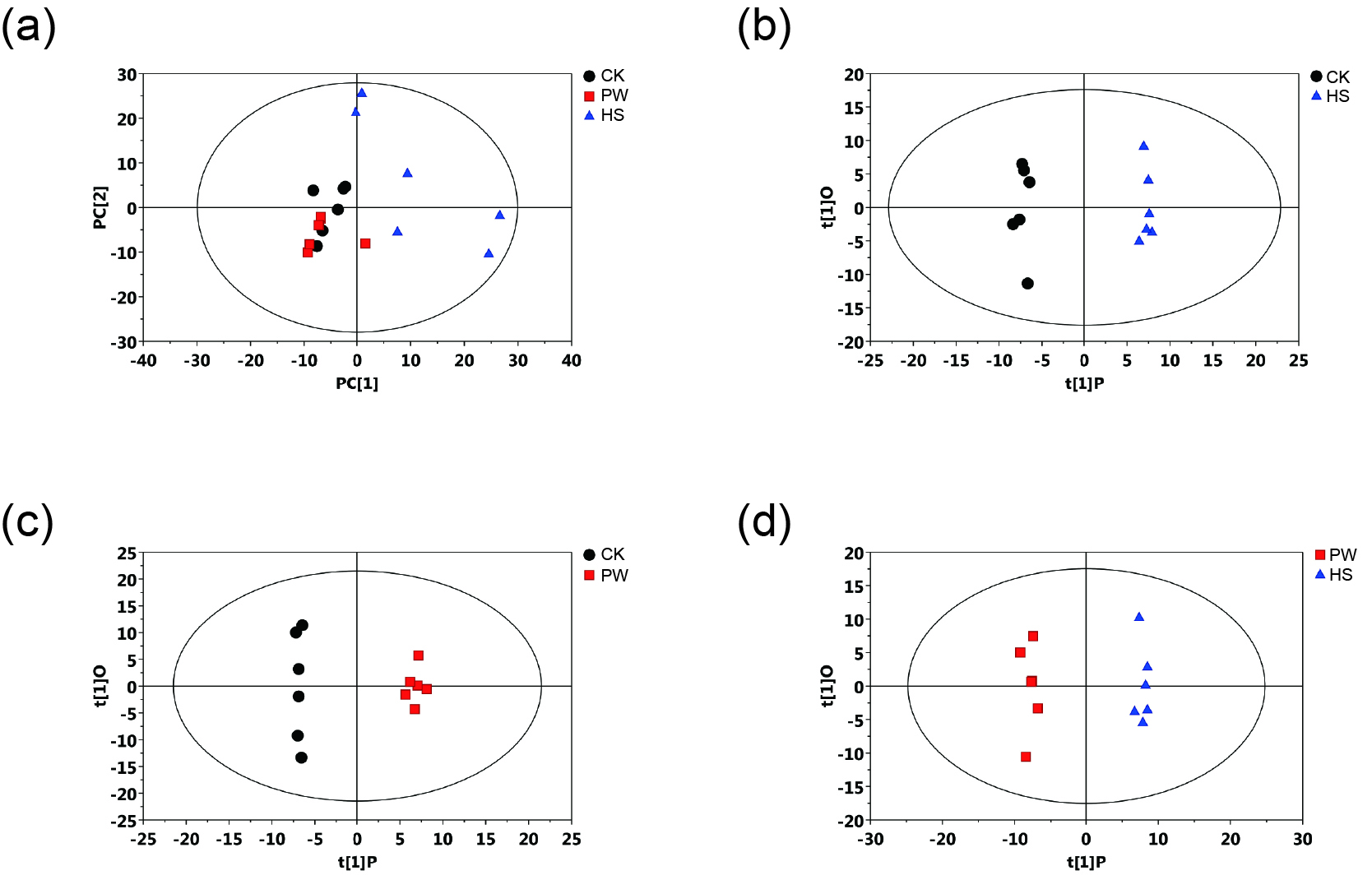

Supplement: Supplementary file 1 — Additional files 1: Figure S1. Principal component analysis (PCA) and Orthogonal projection to latent structure with discriminant analysis (OPLS-DA) of Metabolomic analyses. (a) PCA scores plot. (b)-(d) OPLS-DA scores: (b) control (CK) vs heat shock (HS); (c) control (CK) vs prolonged warming (PW); (d) prolonged warming (PW) vs heat shock (HS). Black circle: control; red square: prolonged warming; blue triangle: heat shock. Each treatment contains six biological repeats. [file 12870_2020_2292_MOESM1_ESM.jpg]

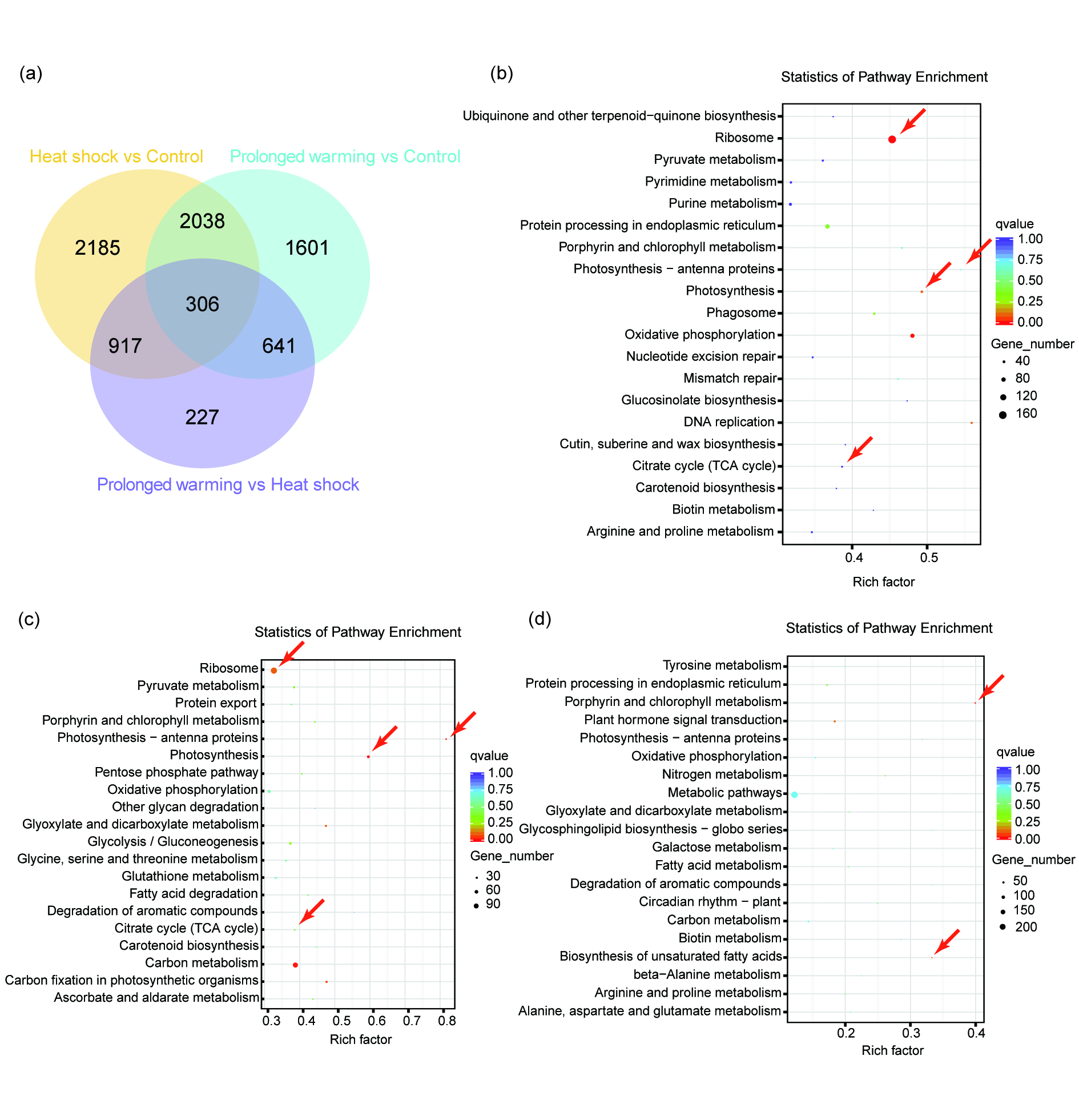

Supplement: Supplementary file 2 — Additional file 2: Figure S2. Gene expression comparisons. (a) Venn diagram of the number of differentially expressed genes (DEGs). Numbers in the overlapping sets show the number of genes that are differentially expression in two or three pairwise comparisons. (b)-(d) Kyoto Encyclopedia of Genes and Genomes (KEGG) pathway enrichment of DEGs. (b) Control (CK) vs heat shock (HS); (c) control (CK) vs prolonged warming (PW); (d) prolonged warming (PW) vs heat shock (HS). The sizes of the dots are in proportion to the number of genes involved. The color of the dot is closer to red as the q-value approaches 0. The genes are considered statisticallysignificantly over-represented, i.e. enriched, when q < 0.05. Red arrows in (b) and (c) indicate those pathway enrichment of DEGs involved in ribosome, antenna proteins, photosynthesis and citrate cycle; Red arrows in (d) indicate the most siginificant enriched pathways. [file 12870_2020_2292_MOESM2_ESM.jpg]

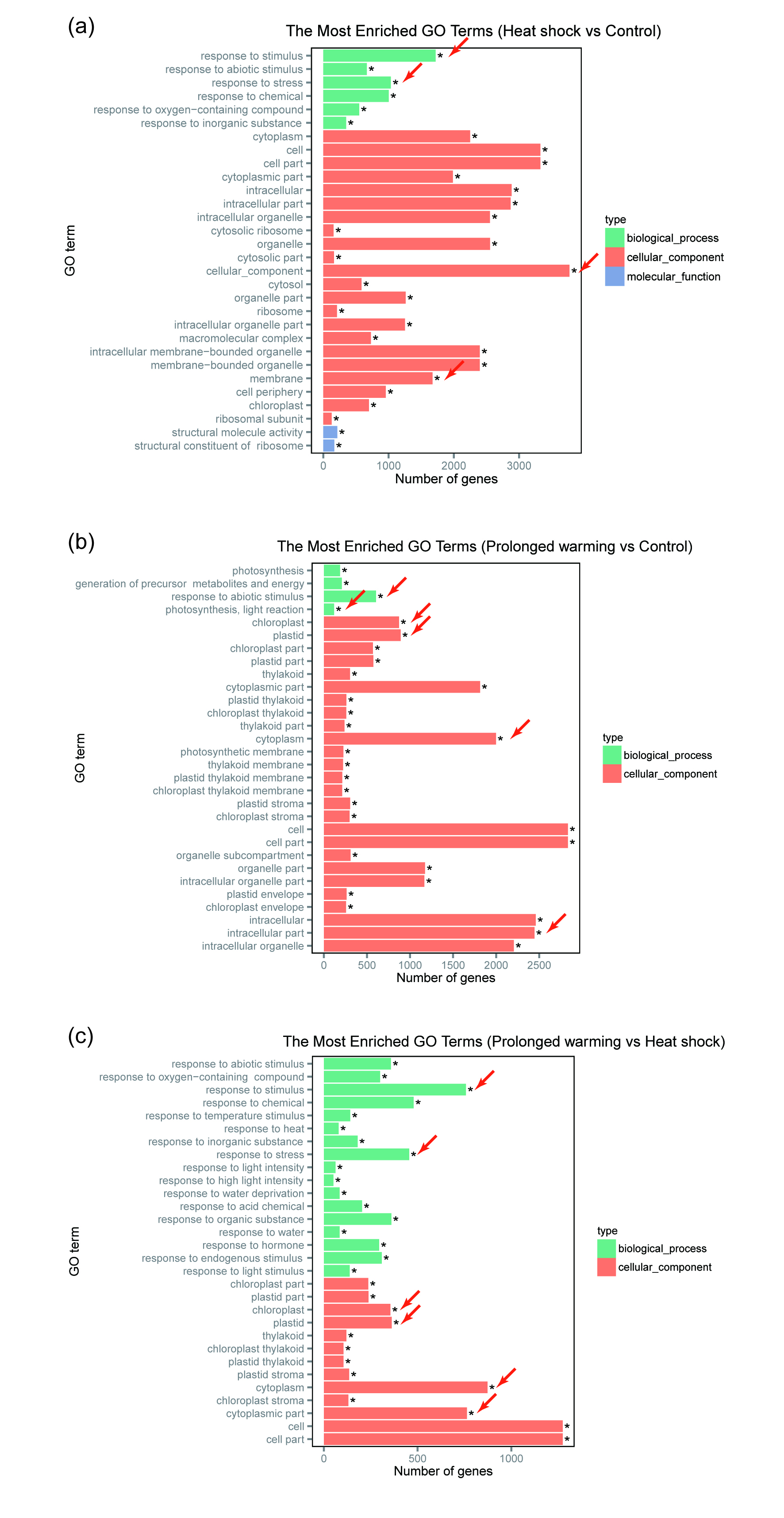

Supplement: Supplementary file 3 — Additional file 3: Figure S3. Gene ontology (GO) term enrichment of DEGs. (a) Heat shock (HS) vs control (CK); (b) prolonged warming (PW) vs control (CK); (c) prolonged warming (PW) vs heat shock (HS) Green bars represent genes involved in biological processes in response to external stimuli, and orange bars represent those involved in the structures of cellular components. [file 12870_2020_2292_MOESM3_ESM.jpg]
