# Supplementary material for: Differential physiological, transcriptomic and metabolomic responses of Arabidopsis leaves under prolonged warming and heat shock
Source: BMC Plant Biol. 2020 Feb 22;20:86. doi: 10.1186/s12870-020-2292-y (PMC7036190; doi:10.1186/s12870-020-2292-y)
Supplement: Supplementary file 4 — Additional file 4: Table S1. Summary of draft reads of samples by Illumina deep sequencing. [file 12870_2020_2292_MOESM4_ESM.docx]

**Table S1**. **Summary of draft reads of samples by Illumina deep sequencing**

| Samples | Total raw reads | Total clean reads | Clean bases | Error rate (%) | Q20 (%) | GC content (%) |
| --- | --- | --- | --- | --- | --- | --- |
| CK | 105740402 | 102596706 | 15.38G | 0.02 | 96.91 | 45 |
| PW | 80726288 | 77761052 | 11.67G | 0.02 | 97.17 | 46.62 |
| HS | 83382386 | 80456340 | 12.07G | 0.02 | 97.11 | 46.25 |
