# Supplementary material for: Differential physiological, transcriptomic and metabolomic responses of Arabidopsis leaves under prolonged warming and heat shock
Source: BMC Plant Biol. 2020 Feb 22;20:86. doi: 10.1186/s12870-020-2292-y (PMC7036190; doi:10.1186/s12870-020-2292-y)
Supplement: Supplementary file 5 — Additional file 5: Table S2. Primers used in qRT-PCR of the leaves of A. thaliana. [file 12870_2020_2292_MOESM5_ESM.docx]

**Table S2.** **Primers used in qRT-PCR of the leaves of *A. thaliana***

| **Gene ID** | **Forward primer (5’–3’)** | **Reverse primer (5’–3’)** |
| --- | --- | --- |
| Actin2 | CTTACAATTTCCCGCTCTGC | GTTGGGATGAACCAGAAGGA |
| HSFA4A | CTGTTGCTGCTCCTCCTCCT | GGTTGAGCCAGGATTCTCT |
| HSFA6A | TTCAATGGAGCATCAAGGACA | CTCCTCCTCACTCAACACGA |
| HSFA6B | ATCGAAGAGGCGATCAGCA | TGAGGATGAGGCTGCAACA |
| DREB2A | AAGCATTGGCTGAGCGAGTT | CCTGCTGTTGTTGCTGACA |
| DREB2C | TGGAGATTGCTCAACCAGGA | CATTGAACCTGCGATGGTC |
| HSFA1A | CGAATATCGGCGAGGCTGTGAC | CGTCGGACTCCATGAGACAATCG |
| HSFA1B | TCATCTGTTGGTGCTTGTGTCGAG | TGCTGTTGCTGCCTTAACCTGAC |
| HSFA1D | AAGAAGCGGAGATTCAAGCGAGAC | TGCTCGTGCATTGGAGGTTGATAC |
| HSFA1E | AGCCTCCACAACAACCTCAAGTTC | TGCTGCCTCAACCTTACAAGTTCC |
| HSFA2 | ATCATGGTGTGCTTGTAGCTGAGG | AACGTCATCATCTGCTGCTGTCTC |
| HSP70-3 | CGTGCCAGATTCGAGGAGCTTAAC | TGAGCCACCAACAAGGACAACATC |
| HSP70-9 | AGGACTGCTATGGCTGGTGAGG | CTCCGATCTTCGACACTGCCTTG |
| HSP70-14 | CGAGAATTGCCTTGTTGCCGTTG | CACCGAAGCAGACAATAGCAGGAG |
| HSP90-2 | GGACTCACCGTGCTGTCTTGTAAC | ACTTGTCGTTCTTGTCTGCGTCAG |
| HSP90-3 | CATGGAGGCGTTGGCTGCTG | ACTGCTCATCGTCATTGTGCTTGG |
| HSP90-4 | GCCGAGAACAAGGAGGACTACAAC | TGGTAACGGAGCAACTCAGCAATC |
| HSP22.0 | TGCTATCCGATCTCTGGCTAGACC | GGAGACAGAGCCACGCTTGTG |
| APX2 | GGTGCCACAAGGAGCGTTCAG | AAGAGCCTTGTCGGTTGGTAGTTG |
| AT1G19490 | GTTACACTGATGCCGCCTGGTC | CCGACCTGCATCCGACATTGAC |
| bZIP9 | CACCATCACACTCCATCAGCAGTC | CCAGATGTCTGAGACGCAGCTAAC |
| bZIP24 | GTGAAGCGGCAAGAGTGGAGTG | CGTGAGAATGGTGGTGGTTGAGG |
| bZIP34 | CCGACGACGACAACCTTCATAGC | ATTGGACGGCGTGGATGTGTTC |
| bZIP61 | AATAGCCTTAGCGACGACGACAAC | GATTCGCCGACGGACCATCTTG |
| bZIP63 | CTCCAGACACTACAAGCAGCCAAG | CCAAGCTCTCAACTCTACGCATCG |
